# Effects of Elevated CO_2_ on Litter Chemistry and Subsequent Invertebrate Detritivore Feeding Responses

**DOI:** 10.1371/journal.pone.0086246

**Published:** 2014-01-22

**Authors:** Matthew W. Dray, Thomas W. Crowther, Stephen M. Thomas, A. Donald A’Bear, Douglas L. Godbold, Steve J. Ormerod, Susan E. Hartley, T. Hefin Jones

**Affiliations:** 1 Cardiff School of Biosciences, Cardiff University, Cardiff, United Kingdom; 2 School of Forestry and Environmental Studies, Yale University, New Haven, Connecticut, United States of America; 3 Institute of Forest Ecology, University of Natural Resources and Life Sciences (BOKU), Vienna, Austria; 4 York Environmental Sustainability Institute, University of York, York, United Kingdom; North Carolina State University, United States of America

## Abstract

Elevated atmospheric CO_2_ can change foliar tissue chemistry. This alters leaf litter palatability to macroinvertebrate detritivores with consequences for decomposition, nutrient turnover, and food-web structure. Currently there is no consensus on the link between CO_2_ enrichment, litter chemistry, and macroinvertebrate-mediated leaf decomposition. To identify any unifying mechanisms, we presented eight invertebrate species from aquatic and terrestrial ecosystems with litter from *Alnus glutinosa* (common alder) or *Betula pendula* (silver birch) trees propagated under ambient (380 ppm) or elevated (ambient +200 ppm) CO_2_ concentrations. Alder litter was largely unaffected by CO_2_ enrichment, but birch litter from leaves grown under elevated CO_2_ had reduced nitrogen concentrations and greater C/N ratios. Invertebrates were provided individually with either (i) two litter discs, one of each CO_2_ treatment (‘choice’), or (ii) one litter disc of each CO_2_ treatment alone (‘no-choice’). Consumption was recorded. Only *Odontocerum albicorne* showed a feeding preference in the choice test, consuming more ambient- than elevated-CO_2_ birch litter. Species’ responses to alder were highly idiosyncratic in the no-choice test: *Gammarus pulex* and *O. albicorne* consumed more elevated-CO_2_ than ambient-CO_2_ litter, indicating compensatory feeding, while *Oniscus asellus* consumed more of the ambient-CO_2_ litter. No species responded to CO_2_ treatment when fed birch litter. Overall, these results show how elevated atmospheric CO_2_ can alter litter chemistry, affecting invertebrate feeding behaviour in species-specific ways. The data highlight the need for greater species-level information when predicting changes to detrital processing–a key ecosystem function–under atmospheric change.

## Introduction

Global concentrations of atmospheric carbon dioxide (CO_2_) could more than double by 2100 [1]. Typically, CO_2_ enrichment leads to increased plant photosynthesis, resulting in greater biomass and production [2]. Plant tissue chemistry is typically modified, with decreasing nitrogen concentrations and increasing carbon-nitrogen (C/N) ratios affecting herbivore life-history and feeding responses [3].

Approximately 90% of primary production in forest ecosystems escapes herbivory and forms detritus [4], providing a crucial energy pool that underpins the trophic structure of soils and adjacent freshwaters [5]. The effect of elevated CO_2_ on the chemical composition of green foliar tissues reduces its palatability to detritivores when it falls as litter [6]. In particular, elevated CO_2_ can reduce litter resource quality by decreasing litter nitrogen content [7], [8], subsequently increasing C/N ratios [9], [10]. Increases in structural [6], [8], [9] and defensive [10], [11] compounds have also been reported, along with both increases and decreases in phosphorus concentrations [12], [13]. The potential for rising CO_2_ concentrations to alter litter chemical composition is established, but the consequences for invertebrate-mediated decomposition – an important ecosystem function – remain unclear [14].

Detritivorous macroinvertebrates are functionally important in detritus-based ecosystems, as they are responsible for both comminution and consumption of litter, releasing nutrients for other organisms, such as saprophagous fungi [15], [16]. To maintain optimal body nutrient concentrations, theoretical predictions and empirical evidence suggest that invertebrates can increase feeding rates of reduced-quality material (e.g. [17], [18]), a process known as ‘compensatory feeding’ (as defined by [19]). Despite this, poor quality litter has also been shown to increase handling times [20], while reducing nutrient assimilation, slowing development rates, and increasing mortality [6], [21]. These conflicting responses have resulted from studies focusing on a small number of species (e.g. [13], [18]), which also fail to incorporate aquatic and terrestrial invertebrates, despite differences in detrital accumulation and energy flow between these habitats [22]. A broad-scale study incorporating a range of invertebrate species from different habitats is essential to identify the unifying mechanisms that govern invertebrate feeding responses to elevated-CO_2_ litter.

We investigated the feeding preferences and consumption rates of eight detritivorous macroinvertebrate species presented with *Alnus glutinosa* (Linnaeus) Gaertner (common alder) and *Betula pendula* Roth (silver birch) leaf litter produced under ambient and elevated atmospheric CO_2_. We tested the hypotheses that: (1) CO_2_ enrichment will reduce leaf chemical quality and, given nitrogen-fixing ability in alder, responses will differ by tree species; (2) when presented with a choice between ambient and elevated CO_2_ litter, invertebrates will prefer ambient material due to its higher quality; (3) when given litter of one CO_2_ treatment only, consumption of elevated-CO_2_ litter will be greater, to compensate for its reduced quality.

## Methods

### Leaf Litter Preparation

**Figure 1:**
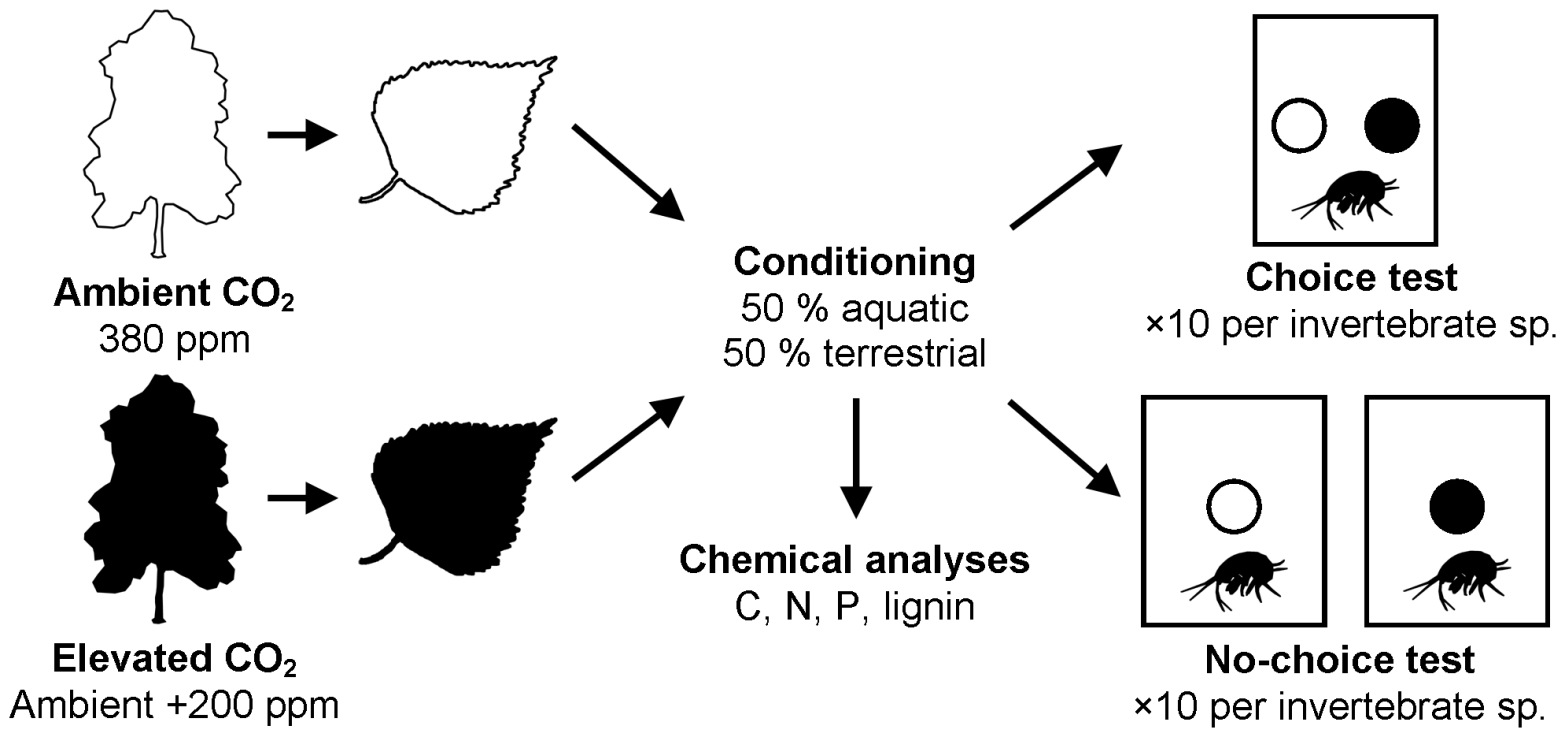
Overview of the experimental approach. Litter was produced under ambient- and elevated-CO_2_ atmospheres at BangorFACE, UK. Half of the litter from each CO_2_ treatment was conditioned aquatically and half terrestrially. Chemical analyses of the conditioned litter were undertaken, and litter discs were presented to aquatic and terrestrial invertebrates in choice and no-choice tests. Only one tree and one invertebrate species have been shown for clarity. Not to scale.

Alder and birch litters were produced at the BangorFACE facility, Bangor, UK [23] (Fig. 1). Trees were grown in eight identical plots (four ambient-CO_2_ and four elevated-CO_2_) to minimise infrastructure-induced artefacts. CO_2_ enrichment was carried out using high velocity pure CO_2_ injection, controlled using equipment and software modified from EuroFACE [24]. Elevated CO_2_ concentrations, measured at 1 min intervals, were within 30% deviation from the pre-set target concentration of 580 ppm CO_2_ (ambient +200 ppm) for 75–79% of the photosynthetically-active period (daylight hours from budburst until leaf abscission) of 2005–2008. Vertical profiles of CO_2_ concentration measured at 50 cm intervals through the canopy showed a maximum difference of +7% from reference values obtained at the top of the canopy [23]. From the beginning of leaf senescence, fallen leaf litter was collected weekly until all leaves had abscised (October to December). Litter within each CO_2_ treatment was homogenised and air-dried.

Initial chemical leaching and microbial colonisation of litter (‘conditioning’) are crucial steps in making litter palatable to detritivorous macroinvertebrates [25], [26]. Prior to the start of the experiment, litter was conditioned in fine mesh bags (100 µm to permit microorganisms only) placed in plastic containers (29×29×10 cm; Fig. 1). For each tree species ×CO_2_ treatment combination, one bag was placed in aerated stream water that was inoculated with stream-collected litter of mixed-species origin (‘aquatic conditioning’); a second bag per tree species ×CO_2_ treatment combination was inserted between field-collected soil and mixed deciduous leaf litter (‘terrestrial conditioning’). Containers were maintained at 11±1°C with a 12∶12 h light-dark cycle and terrestrial containers were sprayed with deionised water every three days to maintain humidity (∼50%). These conditions were selected to represent natural conditioning processes in aquatic and terrestrial habitats in a controlled manner. After two weeks, leaf discs were cut using a 9 mm diameter cork-borer (avoiding the mid-vein), which were air-dried and weighed (±0.1 mg) prior to experimental use.

Litter samples allocated to chemical analyses (Fig. 1) were stored at –80°C before being oven-dried (50°C for 24 h) and ground into powder (120 s, 50 beats s^–1^; Pulverisette 23 ball mill, Fritsch GmbH, Idar-Oberstein, Germany). Each sample was composed of litter from three separate leaves. For carbon, nitrogen and phosphorus analyses, five samples were processed per tree × CO_2_ treatment × conditioning type combination; for lignin analysis, four samples were used. The percentage leaf dry mass (% leaf DM) of carbon and nitrogen, and the carbon-nitrogen (C/N) ratio, were determined by flash combustion and chromatographic separation of ∼1.5 mg leaf powder using an elemental analyser (Elemental Combustion System 4010 CHNS-O Analyzer, Costech Analytical Technologies, Inc., Milan, Italy), calibrated against a standard (C_26_H_26_N_2_O_2_S). Phosphorus concentrations (% leaf DM) were quantified using X-ray fluorescence (see [27] for detailed methodology). The percentage Acetyl-Bromide-Soluble Lignin (% ABSL) was determined following the acetyl bromide spectrophotometric method [28]. Lignin-nitrogen (lignin/N) ratios were calculated for each tree species × CO_2_ treatment × conditioning treatment combination.

### Invertebrates

Eight macroinvertebrate species were selected for study (Table 1), representing a taxonomic range of litter consumers found in temperate forest habitats [29], [30]. Aquatic species were collected from streams in the Brecon Beacons National Park, South Wales, UK (51°50′53″N, 3°22′16″W and 51°50′55″N, 3°33′43″W) and Roath Park, Cardiff, UK (51°30′00″N, 3°10′10″W); terrestrial species were collected from soil-litter interfaces in Bute Park, Cardiff, UK (51°48′49″N, 3°18′24″W). The National Park Authority granted general permission to access sites on common land in the Brecon Beacons National Park, South Wales, UK. Cardiff Council granted permission for access to sites in Cardiff, UK. No endangered or protected species were involved in collections from the field. All individuals were adults, apart from larval *Odontocerum albicorne* and *Sericostoma personatum* caddisflies. Individuals from within each species were selected for size similarity. Prior to experimental use, invertebrates were maintained for at least four weeks in single-species containers (11±1°C, 12∶12 h light-dark cycle) and were fed *Fagus sylvatica* Linnaeus (common beech) litter conditioned as for experimental litter, preventing habituation to experimental alder and birch litter. Feeding was ceased two days prior to the experiments to allow for gut clearance.

**Table 1:** Detritivorous macroinvertebrate species used in the study.

Habitat	Name	Authority	Order: Family
Aquatic	*Asellus aquaticus*	(Linnaeus 1758)	Isopoda: Asellidae
	*Gammarus pulex*	(Linnaeus 1758)	Amphipoda: Gammaridae
	*Odontocerum albicorne*	(Scopoli 1763)	Trichoptera: Odontoceridae
	*Sericostoma personatum*	(Kirby & Spence 1826)	Trichoptera: Sericostomatidae
Terrestrial	*Blaniulus guttulatus*	(Bosc 1792)	Julida: Blaniulidae
	*Oniscus asellus*	Linnaeus 1758	Isopoda: Oniscidae
	*Porcellio scaber*	Latreille 1804	Isopoda: Porcellionidae
	*Tachypodoiulus niger*	(Leach 1815)	Julida: Julidae

### Experimental Arenas

All experiments were conducted in 11×16.5×3.5 cm lidded plastic arenas (Cater For You Ltd, High Wycombe, UK) lined with compacted sterilised aquarium gravel (Unipac, Northampton, UK) and were maintained at 11±1°C with a 12∶12 h light-dark cycle. Aquatic microcosms were filled with 400 ml of filtered (100 µm mesh) stream water (circumneutral pH; collected from 51°50′53″N, 3°22′16″W) and aerated through a pipette tip (200 µl Greiner Bio-One) attached to an air-line. Terrestrial microcosms were sprayed with deionised water every three days to maintain moisture content and humidity (∼50%). All arenas were uniquely labeled (‘microcosm ID’). These standardised conditions were chosen to mimic natural habitats, while minimising the availability of supplementary organic material that could act as a confounding resource during the feeding trials.

For litter of each tree species, detritivores were presented with: (i) a choice between ambient- and elevated-CO_2_ material, to provide a direct comparison of detritivore preferences, and (ii) a no-choice situation with each CO_2_ treatment presented on its own, approximating litter consumption in current (ambient-CO_2_) and future (elevated-CO_2_) atmospheric conditions (Fig. 1). In each experiment, ten microcosms were set up for each invertebrate and tree species combination (*n* = 160). A single invertebrate was added to each arena and was placed in the end opposite the airline in aquatic arenas and equidistant to both discs in the choice test. In the choice test, one disc of each CO_2_ treatment was pinned to the centre of the arena, 4 cm apart. Discs were replenished when at least 50% of the existing disc had been consumed. In the no-choice test, half of the microcosms contained one ambient-CO_2_ disc and the other half one elevated-CO_2_ disc, pinned to the centre of the arena. Both experiments ended after 14 days, or when five (50%) of the individuals of a specific species consumed at least 50% of one disc (choice experiment only). For each invertebrate, the total mass of litter consumed was calculated (±0.1 mg). For choice experiment data, this value was divided by the number of days over which the test had taken place.

Additionally, control microcosms were set up to ensure that differences in mass loss between CO_2_ treatments were due to invertebrate activity alone. For each experiment, ten microcosms were set up for each habitat type × tree species combination. Controls for the choice test each contained one disc of each CO_2_ treatment; half of the no-choice control microcosms contained one ambient-CO_2_ disc and the other half contained one elevated-CO_2_ disc. Leaf discs were air-dried and weighed (±0.1 mg) after 14 days and their total mass loss calculated.

### Data Analysis

Statistical analyses were performed separately for alder and birch litter using *R* version 3.0.1 [31]. Data available from http://dx.doi.org/10.6084/m9.figshare.791634. were checked for normality and homogeneity of variance following Crawley [32]; response variables were transformed using Box-Cox power transformations when assumptions were not met (*car* package [33]). Significance was set at α = 0.05 for all analyses.

Two-way analysis of variance (ANOVA) was used to test the main and interactive effects of CO_2_ treatment and microcosm type on each chemical variable (carbon, nitrogen, phosphorus and lignin concentrations, and C/N ratio). Planned contrasts (*lsmeans* package [34]) were used to compare the effects of CO_2_ treatments for each conditioning treatment.

The main and interactive effects of CO_2_ treatment and microcosm type were tested on the mass loss of control discs. Linear mixed-effects models were used to analyse choice control data (*nlme* package [35]), where non-independence of discs sharing the same microcosm was accounted for by including microcosm ID as a random term. The same fixed terms were used to analyse control data from the no-choice test using two-way ANOVA.

In the choice test, litter consumption per day was analysed using linear mixed-effects models (*nlme* package [35]) with the main and interactive effects of CO_2_ treatment and invertebrate species as fixed effects and microcosm ID as a random effect. Planned contrasts were performed to compare consumption of ambient- and elevated-CO_2_ discs within (i) each invertebrate species, and (ii) invertebrate species grouped by habitat of origin (*contrast* package [36]).

In the no-choice test, the main and interactive effects of CO_2_ treatment and invertebrate species on litter consumption were tested using two-way ANOVA. Planned contrasts were performed to test the effects of CO_2_ treatment on disc consumption within (i) each invertebrate species (*lsmeans* package [34]) and (ii) invertebrate species grouped by habitat of origin (*gmodels* package [37]).

## Results

### Litter Chemistry

CO_2_ enrichment altered leaf litter chemistry, but effects differed between tree species. For birch, CO_2_-enriched litter contained lower nitrogen concentrations, and higher lignin concentrations and C/N ratios than ambient-CO_2_ litter (Tables 2 and 3). Litter chemistry varied between conditioning types, with higher carbon concentrations in aquatically-conditioned litter and lower nitrogen concentrations in terrestrially-conditioned litter (Table 2). For both conditioning types, elevated-CO_2_ litter contained lower nitrogen concentrations (aquatic, estimate = 0.76% DM, *P*<0.001; terrestrial, estimate = 1.17% DM, *P*<0.001; Table 3) and higher C/N ratios (aquatic, estimate = 8.31, *P*<0.001; terrestrial, estimate = 10.28, *P*<0.001; Table 3). For alder litter, the effect of CO_2_ treatment was less predictable, with differential responses between conditioning types (Table 2). Elevated CO_2_ increased alder nitrogen concentrations when conditioned terrestrially (estimate = 0.29% DM, *P* = 0.036; Table 3), although there was no concurrent effect in aquatically-conditioned litter (estimate = 0.1% DM, *P* = 0.44; Table 3). No treatment or species effects on litter phosphorus concentrations were observed (Tables 2 and 3).

**Table 2:** ANOVA summary table of main and interactive effects of CO_2_ treatment (CO_2_) and conditioning type (CT) on litter chemistry.

		Carbon		Nitrogen		Phosphorus		Lignin		C/N	
Tree species	Variables	*F*_1,16_	*P*	*F*_1,16_	*P*	*F*_1,16_	*P*	*F*_1,12_	*P*	*F*_1,16_	*P*
Alder	CO_2_	0.6	0.435	1.1	0.305	2.8	0.117	0.04	0.543	1.3	0.271
	CT	0.3	0.577	4.1	0.059	0.2	0.684	0.2	0.673	3.8	0.071
	CO_2_ × CT	1.5	0.241	4.7	**0.045**	0.4	0.387	3.6	0.082	4	0.064
Birch	CO_2_	0.1	0.712	791	**<0.001**	3.1	0.098	4.8	**0.048**	605.3	**<0.001**
	CT	12.1	**0.003**	95	**<0.001**	0.04	0.848	1	0.331	62.5	**<0.001**
	CO_2_ × CT	3.6	0.077	36.4	**<0.001**	0.3	0.566	0.1	0.756	6.8	**0.019**

*P* values <0.05 are emboldened.

**Table 3:** Chemical composition of leaf litter (mean ±1 SEM).

			Chemical composition				Chemical ratios	
Tree species	CT	CO_2_	Carbon(% DM)	Nitrogen(% DM)	Phosphorus(% DM)	Lignin(% ABSL)	C/N	Lignin/N
Alder	Aquatic	Ambient	48.61±0.37a	3.73±0.16a	0.074±0.009a	22.17±2.64a	13.11±0.16a	5.94
		Elevated	48.48±0.25a	3.63±0.091a	0.064±0.009a	19.56±2.74a	13.37±0.36a	5.38
	Terrestrial	Ambient	48.04±0.22a	3.35±0.016a	0.084±0.009a	19.16±1.01a	14.33±0.02a	5.71
		Elevated	48.68±0.40a	3.65±0.026b	0.062±0.01a	24.34±1.14a	13.35±0.10a	6.68
Birch	Aquatic	Ambient	51.22±0.13a	2.54±0.018a	0.09±0.008a	22.10±3.28a	20.17±0.11a	8.7
		Elevated	50.84±0.13a	1.79±0.004b	0.066±0.01a	27.76±1.69a	28.47±0.08b	15.55
	Terrestrial	Ambient	49.86±0.24a	3.08±0.017a	0.082±0.01a	25.09±2.07a	16.19±0.04a	8.15
		Elevated	50.44±0.41a	1.91±0.063b	0.07±0.006a	29.32±1.52a	26.47±0.74b	15.33

Abbreviations: percent dry mass (% DM), percent acetyl-bromide-soluble lignin (% ABSL), conditioning type (CT).

Different lowercase letters indicate significant differences (*P*<0.05) between CO_2_ treatments for each tree species × CT combination.

### Invertebrate Responses

For both tree species in the choice and no-choice control arenas, disc mass loss in the absence of invertebrates was unaffected by CO_2_ treatment and conditioning type (*P*>0.05). Litter mass loss in the presence of invertebrates was therefore assumed to be a result of invertebrate feeding alone.

**Figure 2:**
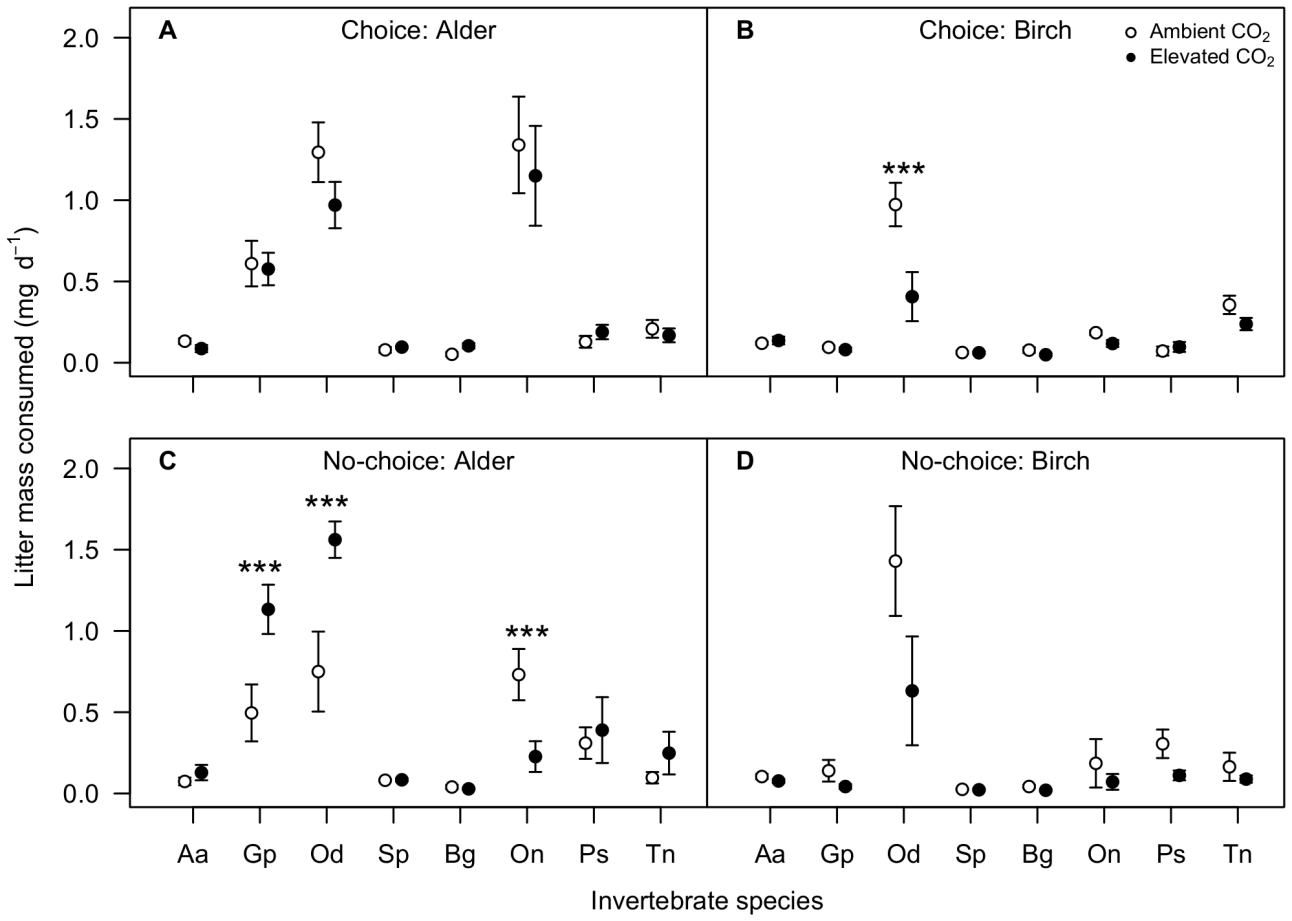
Effects of CO_2_ treatment on feeding responses of each invertebrate species. The mean litter consumption (±1 SE) of each invertebrate species is shown for (A) alder and (B) birch in the choice test, and (C) alder and (D) birch in the no-choice test. Asterisks indicate significant differences between CO_2_ treatments within each invertebrate species (***P<0.001). Species are arranged by habitat of origin: aquatic species are *Asellus aquaticus* (Aa), *Gammarus pulex* (Gp), *Odontocerum albicorne* (Oa) and *Sericostoma personatum* (Sp); terrestrial species are *Blaniulus guttulatus* (Bg), *Oniscus asellus* (On), *Porcellio scaber* (Ps) and *Tachypodoiulus niger* (Tn).

In the choice test, leaf palatability affected invertebrate feeding, but this was dependent on tree species. Birch litter consumption was higher for ambient- than elevated-CO_2_ discs overall (*F*_1,72_ = 10.48, *P* = 0.002); there was no effect of CO_2_ on consumption of alder discs (*F*_1,72_ = 187.21, *P* = 0.34). Consumption also varied between invertebrate species (alder, *F*_7,72_ = 0.92, *P*<0.001; birch, *F*_7,72_ = 30.05, *P*<0.001). The effect of CO_2_ on birch consumption varied by invertebrate species (*F*_7,72_ = 3.44, *P* = 0.003), where *O. albicorne* preferred ambient-CO_2_ discs (estimate = 1.29 mg d^−1^, *P*<0.001; Fig. 2B). The effect of CO_2_ on litter preference did not vary between invertebrates feeding on alder (*F*_1,72_ = 0.5, *P* = 0.83; Fig. 2A). When grouped, aquatic species preferred ambient-CO_2_ birch discs over those grown under elevated CO_2_ (estimate = 1.09 mg d^−1^, *P* = 0.008), but no other preferences were exhibited (aquatic species fed alder, estimate = 0.02 mg d^−1^, *P* = 0.585; terrestrial species fed alder, estimate = 0.03 mg d^−1^, *P* = 0.496; terrestrial species fed birch, estimate = 0.06 mg d^−1^, *P* = 0.061).

In the no-choice test, consumption rates were higher when invertebrates fed on ambient- rather than elevated-CO_2_ birch discs (*F*_1,64_ = 6.39, *P* = 0.014). The trend was consistent across all invertebrate species, but no individual species showed a significant response (CO_2_ treatment × invertebrate species: *F*_7,64_ = 0.341, *P* = 0.932; Fig. 2D). This overall effect of CO_2_ did not occur in alder leaves (*F*_1,64_ = 3.6, *P* = 0.062), but the effect of CO_2_ varied significantly between species (*F*_7,64_ = 4.56, *P*<0.001); more of the elevated-CO_2_ discs were consumed by *G. pulex* (estimate = 2.89 mg, *P* = 0.002) and *O. albicorne* (estimate = 3.22 mg, *P*<0.001), while *O. asellus* consumed more of the ambient-CO_2_ discs (estimate = 2.86 mg, *P* = 0.0022; Fig. 2C). When grouped by habitat, aquatic invertebrates ate more elevated-CO_2_ than ambient-CO_2_ alder (estimate = 1.965 mg, *P*<0.001) but there was no effect on birch (estimate = 0.1 mg, *P* = 0.073). CO_2_ treatment had no effect on consumption by terrestrial species fed either alder (estimate = 0.22 mg, *P* = 0.306) or birch (estimate = 0.1 mg, *P* = 0.085).

## Discussion

Elevated atmospheric CO_2_ and microbial conditioning type modified leaf litter chemistry, though effects differed between tree species (supporting Hypothesis 1). Individual invertebrate species varied in their responses, suggesting that caution has to be taken when extrapolating general trends from single-species studies.

Elevated atmospheric CO_2_ reduced birch litter quality: the concentration of nitrogen decreased and the C/N ratio increased, regardless of conditioning type. Most species did not respond to this change; *O. albicorne* was the only species with behaviour that supported Hypothesis 2, showing a strong preference for ambient-CO_2_ litter. Prior work supports this response: Ferreira *et al*. [13] showed that low C/N ratios reduced birch litter consumption by the caddisfly *Sericostoma vittatum* Rambur, while Cotrufo *et al*. [17] found that the woodlouse *P. scaber* preferred high quality (lower C/N ratio and lignin concentration) *Fraxinus excelsior* Linnaeus litter grown under ambient CO_2_. Alder litter showed negligible chemical change as a result of elevated CO_2_, perhaps due to symbiosis with nitrogen-fixing bacteria that help maintain nutrient supplies [38]. Unexpectedly, a slight increase in quality (increased nitrogen concentration) under elevated CO_2_ occurred when alder litter was conditioned terrestrially, but this did not result in any feeding preferences. Effects of conditioning type on litter chemistry may have occurred due to differences in chemical leaching and microorganism activity between aquatic and terrestrial environments [39]. Our data indicate that CO_2_ enrichment will affect litter palatability to macroinvertebrate detritivores as a result of chemical change, though these effects will be plant and invertebrate species-specific.

In the no-choice test, invertebrates were expected to compensate for low-quality litter by increasing consumption relative to high-quality litter. In contrast to this expectation, compensatory feeding was not observed in either tree species. There was no clear pattern for alder; invertebrate responses were highly idiosyncratic, with *O. asellus* being the only species to consume more of the low-quality resource (terrestrially-conditioned alder litter contained lower nitrogen when grown under ambient-CO_2_). Hättenschwiler *et al*. [18] detected a similar compensatory response for *O. asellus* and another woodlouse, *P. scaber*: higher consumption rates were recorded on low-quality, CO_2_-enriched *F. sylvatica* litter (low nitrogen concentration, high C/N ratio). The current study showed that *G*. *pulex* and *O. albicorne* consumed more elevated-CO_2_ than ambient-CO_2_ alder, despite no observed chemical differences. It is possible that elevated CO_2_ reduced litter palatability by altering chemical constituents that were not quantified here, such as secondary metabolites. For example, phenolics and tannins have been shown to be affected by CO_2_ levels [40]. Birch litter responses appeared less idiosyncratic, with no individual species increasing consumption of elevated-CO_2_ litter. These results suggest that litter species identity determines the predictability of invertebrate feeding responses, but that compensatory feeding is not a unifying trend amongst detritivorous macroinvertebrates.

Feeding rates may have varied due to increased handling times associated with low quality birch litter (e.g. [20]), or because of differences in species’ body chemistry and their ability to cope with elemental imbalances with CO_2_-enriched resources [41], [42]. Heterotrophs, such as the detritivores in our study, tend to maintain constant body elemental composition [43] and may alter feeding behaviour to achieve optimum chemical balance. Our results show that individual invertebrate species rarely demonstrated significant responses to CO_2_ treatments in either test. This suggests that although individual species responses appear idiosyncratic, when considered as a whole, the invertebrate community generally shows consistent and predictable behavioural and functional responses to litter chemical changes induced by elevated CO_2_.

Altered consumption of litter by macroinvertebrates will affect energy release from detritus, in turn affecting secondary production, and food-web structure and functioning [5]. Specifically, on the basis of invertebrate responses in our study, mineralisation of carbon and nutrients could slow down in forests dominated by birch or other tree species with similar chemistry. This is reinforced by our observations of high lignin/N and C/N ratios of elevated-CO_2_ birch litter, which are predictors for slow decomposition rates [44]. Conversely, stands containing a lot of alder, or other species with lower C/N ratios, may show little response in terms of detrital processing and nutrient turnover. Differences between tree species make it difficult to predict overall decomposition rates, a task made more difficult by the prevalence of litter mixtures in temperate deciduous forests, which tend to exhibit non-additive decay [45].

Changes to litter quality as a result of elevated CO_2_ may also affect invertebrate community composition, a potentially important determinant of decomposition rates [19]. This could be caused by changes to food selection [46] and increased patchiness of resource quality in litter mixtures on the forest floor [47]. Differential changes to feeding rates may alter competitive dynamics between invertebrate species, with advantages for species whose dietary breadth extends beyond leaf litter, such as *G. pulex* and *S. personatum* [48], [49].

Our study provides, to date, the broadest assessment of detritivorous invertebrate species’ feeding responses to CO_2_-enriched litter, improving our mechanistic understanding of a key ecosystem process in temperate woodland ecosystems. Future elevations of atmospheric CO_2_ are predicted to affect the breakdown of detritus indirectly by reducing leaf litter quality for macroinvertebrate detritivores. The study highlights that this process is highly tree species-specific, and there will be strong responses in some forest stands and minimal effects in others. Identifying the mechanisms governing such ecosystem variation in functional responses to climate change is essential if we are to predict the consequences of elevated CO_2_ for forest carbon dynamics and nutrient cycling at regional and landscape-scales.
